# Impact of growth rate on graphene lattice-defect formation within a single crystalline domain

**DOI:** 10.1038/s41598-018-22512-5

**Published:** 2018-03-06

**Authors:** Hao-Ting Chin, Jian-Jhang Lee, Mario Hofmann, Ya-Ping Hsieh

**Affiliations:** 1 Graduate Institute of Opto-Mechatronics, National Chung Cheng University, Chiayi County, 62102 Taiwan; 2 Institute of Atomic and Molecular Sciences, Academia Sinica, Taipei, 10617 Taiwan; 3 Department of Physics, National Taiwan University, Taipei, 10617 Taiwan

## Abstract

Chemical vapor deposition (CVD) is promising for the large scale production of graphene and other two-dimensional materials. Optimization of the CVD process for enhancing their quality is a focus of ongoing effort and significant progress has been made in decreasing the defectiveness associated with grain boundaries and nucleation spots. However, little is known about the quality and origin of structural defects in the outgrowing lattice which are present even in single-crystalline material and represent the limit of current optimization efforts. We here investigate the formation kinetics of such defects by controlling graphene’s growth rate over a wide range using nanoscale confinements. Statistical analysis of Raman spectroscopic results shows a clear trend between growth rate and defectiveness that is in quantitative agreement with a model where defects are healed preferentially at the growth front. Our results suggest that low growth rates are required to avoid the freezing of lattice defects and form high quality material. This conclusion is confirmed by a fourfold enhancement in graphene’s carrier mobility upon optimization of the growth rate.

## Introduction

Chemical vapor deposition (CVD) is a promising approach to scalably producing a wide range of two-dimensional materials^1^. Unfortunately, there is a large discrepancy between the anticipated and obtained performance of thus grown materials. This deviation is ascribed to the presence of defects that impart undesirable properties onto a material^2^. CVD grown graphene, the most thoroughly investigated 2D material, has shown high concentrations of defects at the grain boundary which deteriorate the carrier transport. With the recent advent of ultra-large grain sizes^3,4^, these defects can be suppressed and the importance of other defect sources increases. Unfortunately, no consensus exists on the source of defects or the dynamics of defect formation even in graphene. Several reports claim that CVD graphene is inherently perfect and defects only occur at the grain boundaries and the nucleation point^5–8^. This is believed to be due to the large energy difference between perfect and defected graphene which results in a low equilibrium concentration of defects. However, these conclusions were based on experiments on post-growth defect formation using ion bombardment^9,10^ or radiation^11,12^ and only reveal the thermodynamic stability of defects. To be relevant to the growth process, the kinetics of defect formation has to be considered. *In-situ* TEM growth studies found that defects in graphene are not healed but filled with non-hexagon carbon structures^13^ or line-defects are retained^14^. These observations are in agreement with theoretical studies on the defect formation in carbon nanotubes that found a high activation energy barrier for the formation energy of Stone-Wales-type defects^15,16^ which could suggest the kinetic hindrance of defect healing. Thus, even though defects are not thermodynamically stable, they could still occur because their conversion to undefected graphene proceeds too slowly. Recent experimental work on the varying shape and defectiveness of graphene grains supports this hypothesis^17^.

Traditionally, the kinetics of defect formation is investigated through temperature-dependent growth studies. The complex growth process of carbon nanostructures^18^, however, makes this approach challenging. Experimental work on the growth of defected nanotubes, for example, demonstrated multiple defect formation pathways with temperature-dependent activation energy barriers^19^.

To overcome these issues, we here investigate the defect formation during graphene growth at constant temperature but under variable growth rates. This new approach was realized by growing graphene in confinements of varying dimensions. Statistical analysis of the defectiveness by Raman spectroscopy thus allowed us to experimentally investigate the kinetics of graphene defect formation. The defect concentration was found to significantly increase with increasing growth rate. These findings agree with a model of competing defect healing and freezing for outgrowing graphene. The extracted unhealed defect concentration is three times larger than the equilibrium defect concentration which indicates the importance of growth optimization. Using these findings, a fourfold enhancement of graphene’s carrier mobility was achieved.

## Results and Discussion

**Figure 1:**
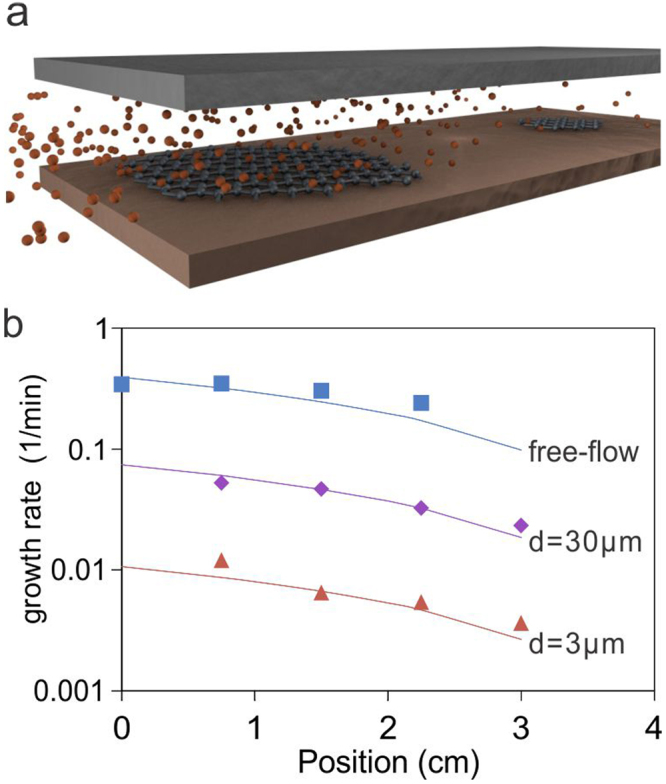
(**a**) Illustration of confinement process resulting in spatially varying precursor concentration and graphene growth rate, (**b**) growth rate vs. position for different gaps and its fitting to the model described in the text

We have previously demonstrated that the growth rate of graphene can be controlled over a wide range by confinement effects as shown in Fig. 1a^20^. Graphene’s growth rate in the gap between a copper foil and a dielectric cap was found to depend on the dimension of the gap and the position within such a pore **(**Fig. 1b**)**. This behavior could be ascribed as a transport limited growth process in which growth rate k(x) depends on the diffusion coefficient D and the edge growth rate k(0) according to:

1 $$k(x)=k(0)-Dx$$

The diffusion coefficient D obeys the Bosanquet equation and thus scales with gap size d as shown below:

2 $$\frac{1}{{\rm{D}}({\rm{d}})}=\frac{1}{{D}_{free}}+\frac{1}{{D}_{Kn}}=\frac{1}{{D}_{free}}(1+\frac{\lambda }{d}),$$

where λ is the mean free path of carbon precursors which was previously extracted to be approximately 100 µm^20^.

The edge growth rate k(0) was found to be proportional to D because of sufficient rarefication at the investigated growth conditions. It was found that the growth rate within confinements could thus be predicted using a simple model^20^:

3 $$k(x,d)=D(d)(\frac{4.0\,{n}_{total}}{{n}_{0}}-x),$$

where n_0_ is the carbon concentration at reference conditions (9 Torr, 1000 °C).

The fitting of this simple model with experimentally extracted growth rates shows good agreement over a wide range **(**Fig. 1b**)** and supports our approach to vary gap size and sample position to modify the growth speed.

**Figure 2:**
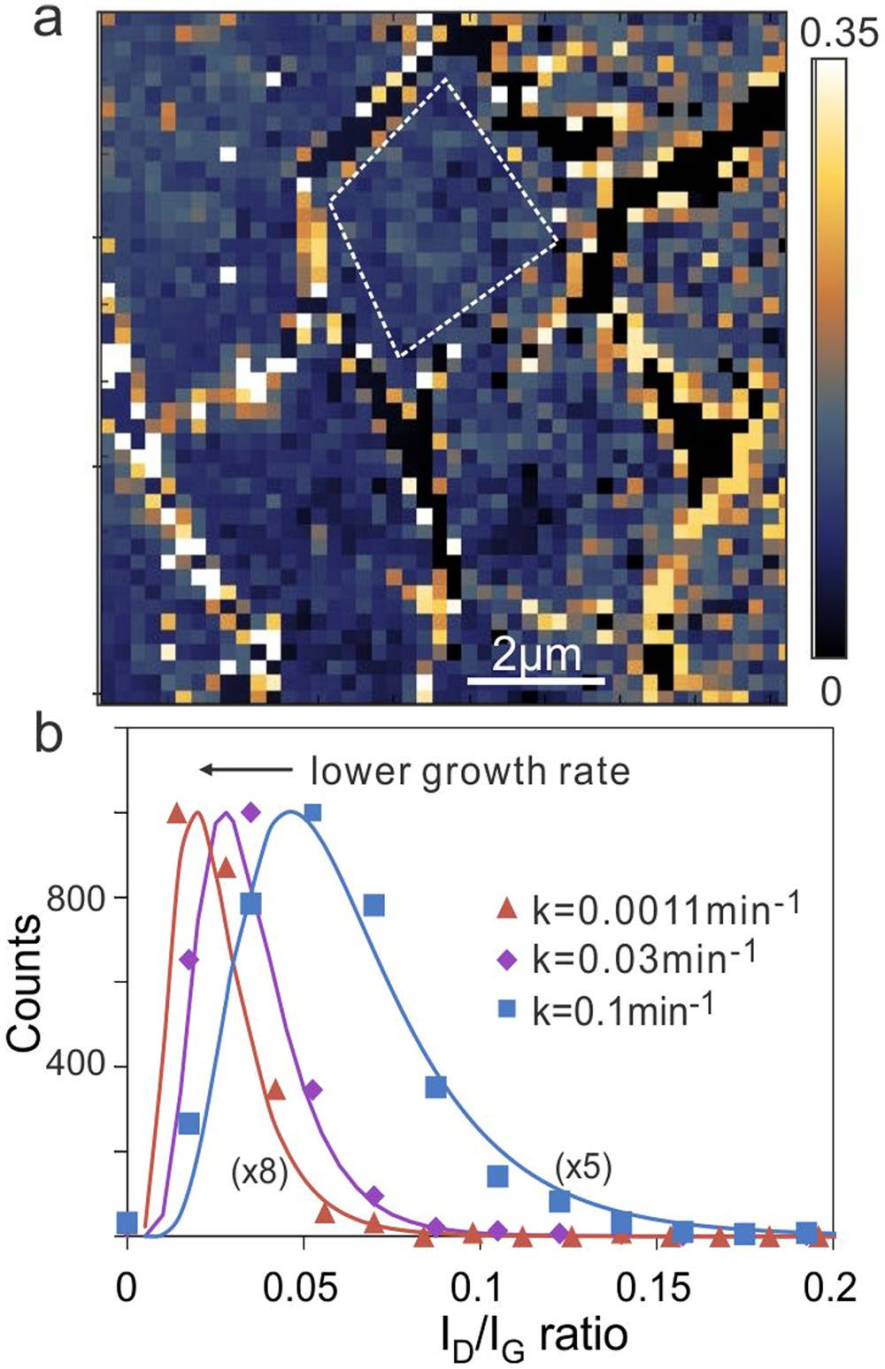
(**a**) Representative map of the Raman I_D_/I_G_ ratio for graphene grown in a pore with 3 µm gap size with indication of area used for further analysis, (**b**) representative I_D_/I_G_ distributions for three growth rates.

To investigate the defectiveness of graphene grown at thus modified growth rates we employ spatially resolved Raman spectroscopy due to its higher sensitivity to low concentration defects compared to microscopic characterization techniques^21^. Figure 2a shows a representative map of the defect-related Raman I_D_/I_G_ ratio. We observe a higher defect concentration at the grain boundaries in agreement with previous reports^7^. Since the boundaries are not representing the pristine graphene lattice, we disregarded them from further analysis by visually identifying grains and selecting regions of interest within the center of such grains. Multiple grains are analyzed within a Raman map and their specific I_D_/I_G_ ratios are summed up to provide statistics for a single sample location or growth rate.

The obtained mapping data was then used to produce a histogram of observed I_D_/I_G_ ratios **(**Fig. 2b**)**. We find an asymmetric distribution of I_D_/I_G_ ratios with a tail towards higher defectiveness that had been previously attributed to the modification of metastable bonds in graphene under laser irradiation during mapping^18–22^. It had been suggested that the observed defect distribution is a product of the original defect concentration and a time-dependent increase in defect concentration during Raman mapping. Thus, the center and extend of the distribution are affected by the initial defectiveness. By fitting each Raman I_D_/I_G_ histogram to a lognormal distribution, we can extract its mode (m) which represents a measure of the graphene’s initial defectiveness when comparing Raman maps that were taken under identical irradiation conditions.

When correlating graphene’s growth rate with the I_D_/I_G_ distribution, we measure a threefold decrease in both distribution mode and distribution width for graphene grown under identical growth conditions by adjusting gap size and sample position. We thus hypothesize that samples with lower growth rates produce a narrower I_D_/I_G_ distribution with fewer defects. Theoretical work had predicted an effect of growth rate on defectiveness during defect healing in carbon nanotube walls^16^. Simulations suggest that defect healing will be slower when they are surrounded by more carbon atoms and efficient defect healing would only occur at the front of the outgrowing wall. Thus, the activation energy barrier for defect healing will increase with separation between the nanotube’s growth front and the defect and if the growth rate exceeded the timescales required for defect healing, lattice defects could remain. Based on the described model, the defect concentration (C) scales with growth rate (k) according to^16^

4 $$C={C}_{eq}+({C}_{0}-{C}_{eq}){\exp }(-\frac{kT}{h}({\exp }(-\frac{{E}_{r}}{kT})+{\exp }(-\frac{{E}_{r}+{E}^{\ast }}{kT})\,)\frac{\Delta h}{k}\,){\boldsymbol{,}}$$

where ${{\rm{C}}}_{{\rm{eq}}}$ is the equilibrium defect concentration for infinitely slow growth and ${{\rm{C}}}_{0}$ is the unhealed defect concentration. The ${{\rm{E}}}_{{\rm{r}}}$ and ${{\rm{E}}}_{{\rm{r}}}+{{\rm{E}}}^{\ast }$ represent the energy barriers for healing and formation of defects. Finally, Δh is the distance between defect and growth front.

**Figure 3:**
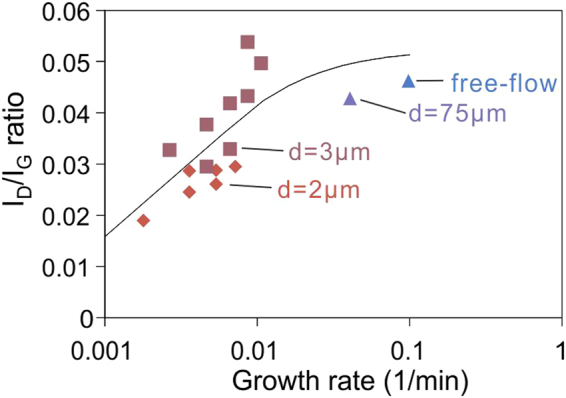
Mode of I_D_/I_G_ distributions vs growth rate with fit according to equation 1.

To investigate the applicability of the model to graphene defect formation, we conducted Raman mapping in 16 different graphene samples that had been grown at different locations within gaps of different sizes. We observe a clear trend between the growth rate at a sample’s position and its defectiveness for all investigated conditions **(**Fig. 3**)**.

We proceed to fit our defectiveness vs. growth rate data to equation 4 by treating all other variables as fitting parameters and observe a good agreement between experimental results and the model **(**Fig. 3**)**. These results allow several important observations of the graphene growth process. First, at free-flow conditions the defect concentration approaches the unhealed defect concentration indicating that such growth conditions are too fast to produce high quality graphene. Secondly, slowing down of the growth rate yields an equilibrium condition that is approximately three times smaller. We can thus conclude that defect healing is an important and necessary step during graphene formation.

**Figure 4:**
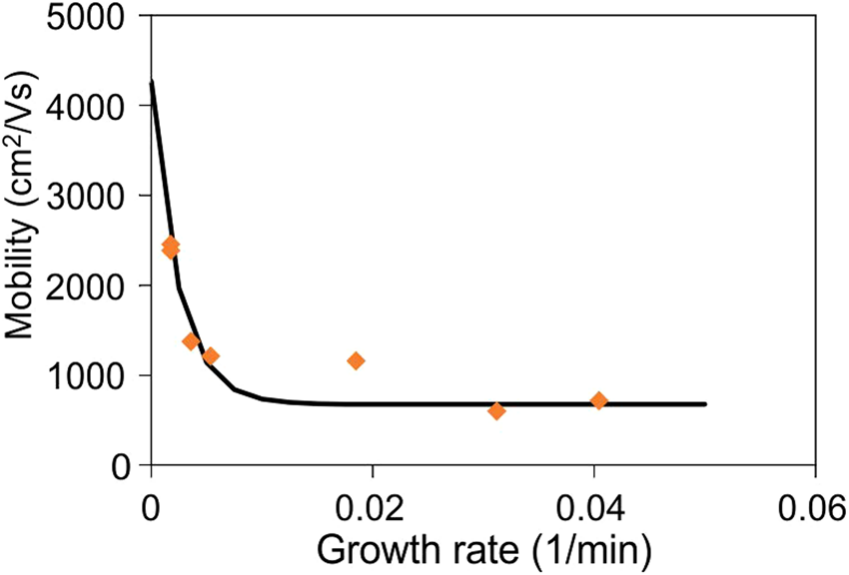
Hall mobility vs. growth rate.

To prove the validity of our analysis, we investigate the carrier transport of graphene samples grown at different growth rates. Figure 4 shows a clear decrease in graphene’s mobility with increasing growth rate which supports the results obtained by Raman characterization. Interestingly, the extracted mobility tends to a finite value of ~4200 cm^2^/Vs for infinitely slow growth. This behavior is due to the finite equilibrium concentration of defects that cannot be healed which is in agreement with the non-zero intercept in Fig. 3. The Raman I_D_/I_G_ ratio for the equilibrium defect concentration is approximately 1.5% which is comparable to the values of exfoliated graphene^23^.

While these results indicate the high quality of graphene grown by CVD, our analysis also reveals a strategy for further enhancing its quality. An energy difference of −0.43 eV was extracted between defected and healed graphene. This value is significantly lower than theoretical predictions for other catalytic materials, such as Fe (−1.5 eV)^16^. Thus, at the investigated growth conditions, an improvement in defect concentration by 4 orders of magnitude could be attained if other catalysts were employed.

## Conclusion

In conclusion we have provided direct measurement of the defect formation kinetics in graphene. We find a clear correlation between growth rate and defect concentration that indicates the importance of defect healing during graphene growth. Based on this finding, we show a four-fold increase in graphene carrier mobility by decreasing the growth rate. These findings suggest a tradeoff between quality and growth speed which has to be considered for the scalable production of graphene.

## Methods

Graphene was synthesized on large pieces (2 × 7 cm) of copper foil (99.8%, Alfa-Aesar, no. 46365) following previous reports^20^. Briefly, Cu was electropolished and annealed under a flow of 200 sccm H_2_ at 1000 °C and 9 Torr for 30 minutes before 10 sccm CH_4_ was introduced to initiate graphene growth.

Pores were produced by inserting suitable refractory spacers between the growth substrate and a refractory cap **(**Fig. 1a**)**. For various sized pores, spacers with different sizes were inserted and for free flow growth, no cap was used.

Investigation of the graphene grain morphology was conducted by optical microscopy (OM) after oxidation of the Cu foil at 200 °C. The growth rate was then extracted by fitting the graphene coverage to equation 5, where k is the growth rate^24^.

5 $$C(t)=100 \% (1-exp(-kt))$$

## Electronic supplementary material

Supplementary material
