# Supplementary material for: Impact of growth rate on graphene lattice-defect formation within a single crystalline domain
Source: Sci Rep. 2018 Mar 6;8:4046. doi: 10.1038/s41598-018-22512-5 (PMC5840368; doi:10.1038/s41598-018-22512-5)
Supplement: Supplementary file 1 — Supplementary material [file 41598_2018_22512_MOESM1_ESM.docx]

Supplementary material

Impact of growth rate on graphene lattice-defect formation within a single crystalline domain

Hao-Ting Chin^1^, Jian-Jhang Lee^2^, Mario Hofmann^3^, and Ya-Ping Hsieh^2*^

1. Graduate Institute of Opto-Mechatronics, National Chung Cheng University, Chiayi County, 62102, Taiwan

2. Institute of Atomic and Molecular Sciences, Academia Sinica, Taipei, 10617, Taiwan

3. Department of Physics, National Taiwan University, Taipei 10617, Taiwan

**Carrier transport in defective graphene**

It was found that the carrier concentration at first increases with growth rate because the increased number of defects provides more anchoring points for gaseous adsorbates such as oxygen that dope graphene. The enhanced electron scattering from those charged impurities is the first mechanism to reduce carrier mobility with defectiveness as shown in Figure 4.

However, the mobility is not only affected by this mechanism as can be seen at higher growth rates. Doping-induced charge scatterers have been found to be randomly distributed and the mobility decrease is cancelled out by the increase in carrier concentration resulting in a constant sheet resistance value^1^. In the high defectiveness regime, however, the sheet resistance is also increasing indicating the modification of the graphene’s intrinsic conductivity^2^.


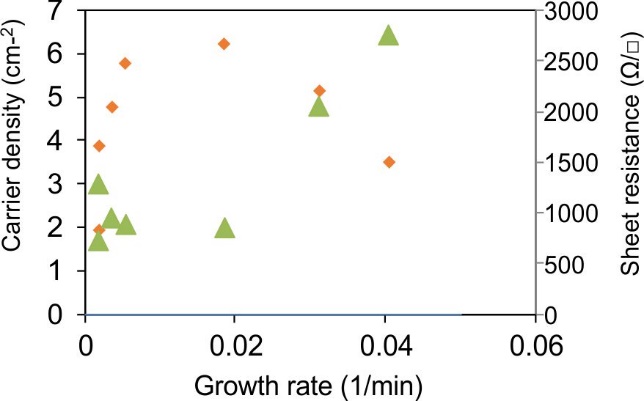


Figure 1 Carrier density extracted by Hall measurements and sheet resistance as a function of growth rate.

**Details on Hall effect measurements**

Hall effect measurements were carried out in Van-der-Pauw geometry at room temperature on cm-scale continuous graphene films. The observed linear trend in the R_xx_/R_xy_ vs. R_xx_ plot indicate the independence of the Hall resistance from the sample resistance and corroborate the high sample quality^2^.


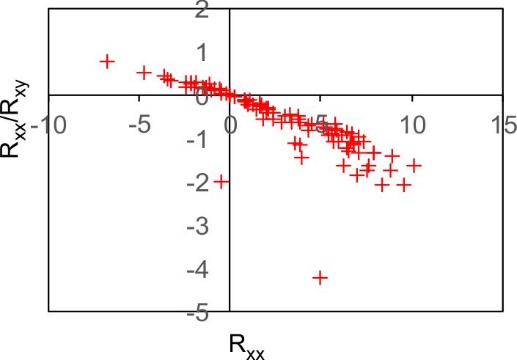


**Film morphology at different growth rates**

Despite large differences in the growth rate, no significant variation in the grain density was observed in agreement with our previous reports^3^ that the employed rarefied conditions decrease the nucleation density towards the intrinsic limit given by the roughness of the substrate. The grain density controls the separation between neighboring grains and thus determines the maximum extend that a grain can grow before it merges. Analysis of optical micrographs of merging grains shown below indeed confirm that the graphene domain size is independent of growth conditions.


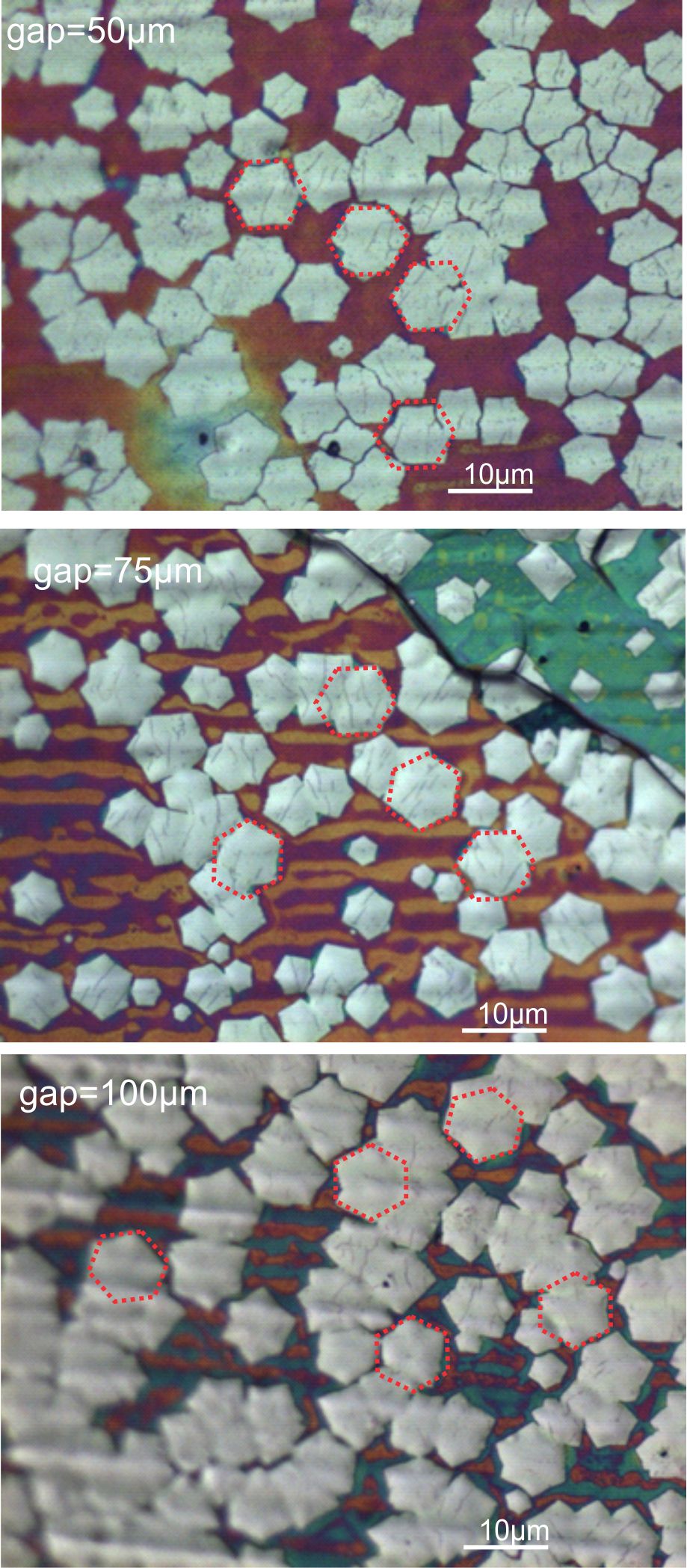


Reference

1 Hsieh, Y. P., Kuo, C. L. & Hofmann, M. Ultrahigh mobility in polyolefin-supported graphene. *Nanoscale* **8**, 1327-1331, doi:10.1039/c5nr07122d (2016).

2 Hofmann, M., Hsieh, Y. P., Chang, K. W., Tsai, H. G. & Chen, T. T. Dopant morphology as the factor limiting graphene conductivity. *Scientific Reports* **5**, 17393, doi:10.1038/srep17393 (2015).

3 Hsieh, Y. P., Shih, C. H., Chiu, Y. J. & Hofmann, M. High-Throughput Graphene Synthesis in Gapless Stacks. *Chem. Mat.* **28**, 40-43, doi:10.1021/acs.chemmater.5b04007 (2016).
